# The Effects of Monetary Incentives on Physicians’ Effort and Patient Satisfaction: Understanding the Links between Monetary Incentives and Physicians’ Effort

**DOI:** 10.3390/ijerph192013075

**Published:** 2022-10-11

**Authors:** Chenhao Yu, Xiaoyan Li, Huigang Liang, Zhiruo Zhang, Dong Fang

**Affiliations:** 1 School of Public Health, Shanghai Jiao Tong University School of Medicine, Shanghai 200025, China; kg.chenhao@gmail.com (C.Y.); lixxiaoyyan104161@sina.com (X.L.); 2 Kunshan Integrated TCM and Western Medicine Hospital, Kunshan 215332, China; 3 Department of Business and Information Technology, Fogelman College of Business and Economics, University of Memphis, Memphis, TN 38152, USA; 4 Shanghai Jiao Tong University-Yale Joint Center for Health Policy, Shanghai Jiao Tong University, Shanghai 200030, China

**Keywords:** monetary incentive, performance pay, patient satisfaction, work performance

## Abstract

How monetary incentives promote physicians’ job performance in terms of patient satisfaction has been widely discussed. The incentive dilemma debate concerns whether monetary incentives reduce physicians’ intrinsic motivation at work and even lead to moral hazard. This study investigated monetary incentive policies in a hospital and analyzed how monetary incentives affect performance and behavior. By means of income composition grouping, a treatment group and control group were established, and the identification of the effect on performance was implemented using the difference-in-difference (DiD) method. The mechanism analysis was implemented using the event-study approach (ESA) and path analysis. The study found that (1) monetary incentives promote physicians to improve patient satisfaction, and the average effect is a two-point increment (*p* < 0.0001); (2) the effects are short-term; and (3) in contrast to many criticisms, the improvement in patient satisfaction was mainly from the effort in working during the monetary incentive policy. The results of this study contribute empirical evidence regarding the effects of monetary incentives and their mechanism and can help hospital management formulate incentive plans.

## 1. Introduction

Patient satisfaction affects outcomes after clinic visits [1], and it is also a predictor of survival for patients during treatment [2]. Therefore, improving patient satisfaction and promoting the level of patient-centered care have become popular issues in healthcare. However, the work pressure and burnout caused by COVID-19 are leading to physician burnout [3], which may reduce the satisfaction of patients [4,5,6]. Thus, for public health and healthcare providers, how to improve the level of patient-centered care is an important question for improving patients’ wellbeing and outcomes. Furthermore, how to motivate medical staff to provide patient-centered medical services and improve patient satisfaction has become a new issue in the context of the pandemic [7].

Limited by the public-health and fiscal policies of various countries, the incentive tools for medical staff are extremely limited. Since improving patient satisfaction is also beneficial to the hospital itself, hospitals also encourage medical staff to improve patient satisfaction through their own human resource policies. However, whether he monetary incentives would enhance physicians’ job performance in terms of patient satisfaction is still unknown [8,9]. One of the core dilemmas is whether monetary incentives are a good choice. The answer concerns how physicians react to such incentives, and how their choice and striving are influenced by monetary incentives [10].

Monetary incentives can provide utility by promoting rational behavior in people [11]. Due to the flexible usage of money, monetary incentives can even be treated as an option, which broadens the effect of the incentives. However, empirical studies have shown very different results. On the one hand, monetary incentives need to be negotiated within the organization, and impairments incurred during the negotiation process can also negatively impact the organization’s long-term performance [12]. On the other hand, the performances of knowledge employees and their contributions are heterogeneous and hard to compare directly; thus, an asymmetric information market has emerged [13], and rational people will tend to maximize key performance indicators. Thus, they have the motivation to cheat, which is why dishonesty is difficult to mitigate [14], and this can sometimes harm long-term performance [15]. An experimental investigation showed that monetary incentives could provide benefits when they complemented relational incentives [16].

**Figure 1:**
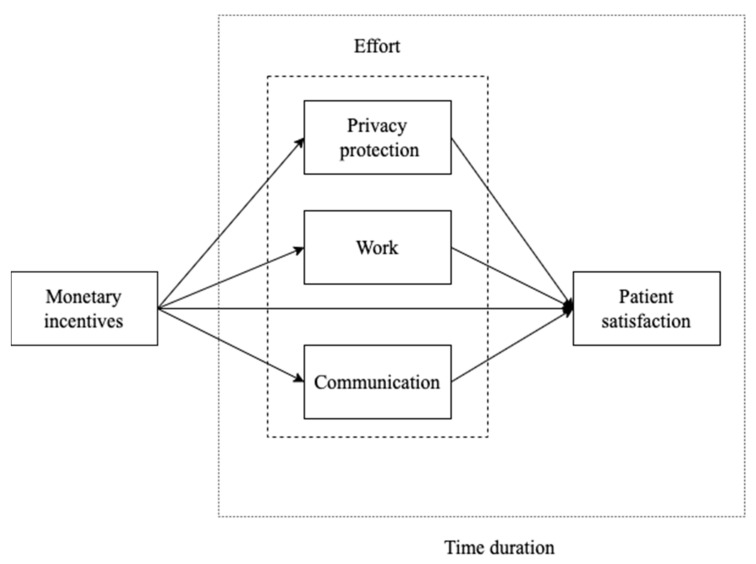
The conceptual framework of the study identifying the effect of monetary incentives on effort and performance.

Although there is considerable heterogeneity between studies, monetary incentives generally improve organizational performance, and what demands our attention is how the adverse effects of monetary incentives can be avoided. To maximize the identification of the effects of monetary incentives and the problems that arise from them, our conceptual framework was modified from Bonner and Sprinkle [17], and our investigation was implemented in a secondary hospital in Jiangsu. For the convenience and feasibility of the intervention, the monetary incentive method used in this study was to reward the department when the patient satisfaction score was greater than 97 points and penalize the department when the patient satisfaction score was lower than 96 points. Rewards and penalties were the same amount and were limited to 5% of the physicians’ average income. The performance was understood in terms of patient satisfaction, which can not only reflect healthcare quality [18] but also provides a proxy of patient compliance, a form of loyalty that can influence clinical outcomes [19]. Effort was considered in four dimensions: communication, working, privacy protection and time duration. The framework of our study is summarized in Figure 1.

Therefore, the research described in this paper can be divided into two parts. The first part involved identifying whether monetary incentives can improve patient satisfaction. The second part involved identifying the path by which physicians make effort after monetary incentives were initiated under our conceptual structure.

## 2. Materials and Methods

### 2.1. Data

We collected panel data provided by patients who had received internal medical, pain treatment, obstetrics, stomatology, pediatrics, surgical or dermatology services from April 2020 to February 2021, and the questionnaire based on the Hospital Consumer Assessment of Healthcare Providers and Systems Survey (HCAHPS^®^). All the data were collected monthly using computer-assisted telephone interviews, and both outpatients and inpatients were randomly interviewed 40 days on average after completion of the medical service. Due to the fact that all the data were collected randomly, the heterogeneity within-group effect was manageable, and the data were treated as repeated measurements of different departments.

### 2.2. Identification Model

As shown in Figure 1, the conceptual framework had three parts. Firstly, we evaluated the effect of policy, and the difference-in-difference method was implemented. Secondly, to ensure the DiD method was set correctly, and to identify the time duration of the monetary incentives, an ESA was implemented. If the model was set correctly, the coefficients before the implementation of incentives should have been zero. The change in the coefficients after the policy indicated the time duration effect. Thirdly, we implemented two-stage regression and the Sobel test to test the effects of different efforts.

#### 2.2.1. Variables and Model

We examined two different forms of patient satisfaction, including for hospitals and physicians. To eliminate case-mixed ratings which result in incomparability among different patient satisfaction questionnaires, we controlled for the age, education, gender and household registration of patients [20]. The fixed effects of departments and time were also considered in the specification model. Thus, our specification model can be written as:

$$Y_{it}=\alpha +\beta \mathrm{DiD}+X\gamma +u_{i}+T_{t}$$

where *Y_it_* is the patient satisfaction for department *i* in month *t* and X is the matrix of control variables. The DiD was 1 when the department was in the treatment group after the monetary incentive plan was executed To implement the DiD estimation, the two variables *treat* and *post* were assigned. The variable *treat* was 1 when the department was classified as a treatment group, and the variable *post* was 1 after the intervention was implemented. Due to the two-way fixed effect applied, the *treat* and *post* variables were omitted in the equation to avoid collinearity. In the process of empirical testing, the respondents were classified according to their medical departments, so this study adopted the cluster-robust standard error as the test statistic. The event-study approach was implemented to verify the parallel trend assumption of the DiD model, and the specification model can be written as:

(1) $$Y_{it}=\alpha +\sum \beta_{\mathrm{it}}\mathrm{Befor}e_{it}*T\mathrm{rea}t_{it}+{\gamma \mathrm{Current}}_{\mathrm{it}}*T{\mathrm{reat}}_{\mathrm{it}}+\sum \delta_{\mathrm{it}}\mathrm{Afte}r_{it}*T\mathrm{rea}t_{it}+u_{i}+T_{t}$$

Here, the core assumption is that the β vector is a zero vector.

Two-stage identification could replace *Y_it_* as physicians’ efforts in work, privacy protection and communication, and the Sobel test [21] was implemented to test the hypothesis of the conceptual framework.

#### 2.2.2. Group Setting

To identify the effect of the incentive plan and implement the DiD method, we extracted the treatment group and control group from the hospital, although the incentive plan was issued to all departments.

To define the control group and the treatment group, the composition of income for physicians in different departments was considered. According to the composition of income, job-level work overload could determine the income of physicians. Due to the zero-markup policy in China, the influence of the amount of income in internal medicine has become very small, because most of the income of internal medicine staff has been blocked [22]. Moreover, the departments were in the control group, for which patients’ medical demands were non-rigid, or the patients played a significant role in treatment-seeking behavior.

The departments where medical staff were motivated to take the initiative to improve patient satisfaction for their income were directly affected by patient satisfaction, and patient satisfaction was the competitive strength in the development of the department. Based on such rules, internal medicine and obstetrics/gynecology were considered as the treatment group. Dentistry and dermatology were considered as the control group (see Table 1 for details).

To ensure the robustness of the group, this paper used the performance bonus/salary as the reference for grouping. We found that the results of the group setting were consistent with the theoretical inferences.

## 3. Results

### 3.1. Summary Statistics

As shown in Table 2, there were 994 respondents. A total of 66% of the respondents were women, the average age of the respondents was 45 years old, 54% of the respondents were residents, and more than half of the respondents had only received junior high school education and below. Hospital and physician satisfaction exceeded 95, and the three effort paths also exceeded 95.

In this study, internal medicine and obstetrics were treated as the treatment group, and dentistry and dermatology were treated as the control group. A total of 84% of the responses were classified into the treatment group.

### 3.2. Empirical Results

Table 3 and Table 4 show the results of the DiD identification. Columns (1)–(4) of the two tables provide the dosage effects of the monetary incentive policy on patient satisfaction and the effort change for communication, working and privacy protection, and the coefficients are represented in the DiD row. After the monetary incentive policy was released, the satisfaction measures of the treatment group all improved. Compared with the intercept, we can conclude that even the baseline of satisfaction was high, and improvement is still possible.

Both of the last columns in Table 3 and Table 4 show how monetary incentives promote patients’ satisfaction. From the physician side, monetary incentives promote (1) the effort in working as much as possible (*p* < 0.0001, Sobel test) to improve patients’ satisfaction, such as through more detailed physical examinations and more careful disease analysis, according to the feedback from patients; (2) the effort in privacy protection (*p* < 0.0001, Sobel test) to improve patients’ satisfaction; and (3) the effort in communication (*p* = 0.0029, Sobel test) to improve patients’ satisfaction, such as by providing more details about the condition, explaining the condition, and providing a more detailed explanation of the physicians’ order.

### 3.3. Event-Study Approach

**Figure 2:**
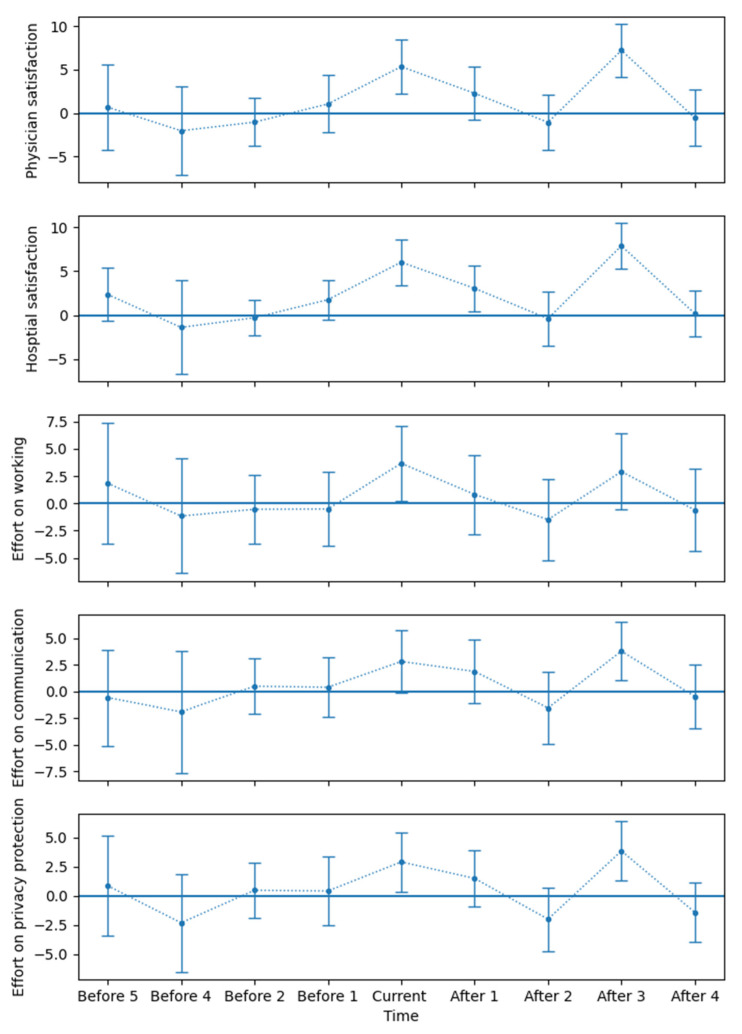
The event-study approach for the effects on physician satisfaction; hospital satisfaction; and efforts in working, commutation and privacy protection. The upper and lower bounds of the error bars are 95% confidence intervals with cluster-robust standard errors clustered by department.

The core assumption of DiD concerns parallel trends, which provide the assurance of the causal relationship reflected by the estimator [23]. Figure 2 shows the results of the event-study approach, and the element released 3 months before the policy was dropped due to collinearity. None of the coefficients were significant under 95% confidential intervals, showing that the assumption of parallel trends was valid.

The coefficients from the monetary incentive policy released also suggested a periodic monetary incentive policy by which the month of payment had the strongest effect, and the effect of other months was not significant. We could also conclude that the monetary incentive policy has a strong short-term effect but not a long-term effect.

## 4. Discussion

In this study, we identified the effect of monetary incentives, and the effect was significant in both statistics and economics. The event-study approach not only tested the parallel trend assumption of the DiD method but also identified that the plan only had a short-term effect, which was significant on payday. In addition, this study examined which types of efforts motivated physicians would make to improve patient satisfaction.

In contrast to previous studies, this study found that the effect of monetary incentives could promote physicians’ efforts in working, communication and privacy protection, and the direct effect and indirect effects were both economically and statistically significant [24]. However, the short-term effect identified in this study also exposed that the medical staff were not interested in improving patient satisfaction or providing patient-centered care, which is worthy of attention [25]. The good news is that, when monetary incentives were implemented, we found that physicians put in more work effort than just pleasing patients, even though nobody informed the physicians how to improve patient satisfaction before and after the implementation of the incentive policies.

In addition, the patients’ perception of physician work may have the highest impact on patient satisfaction from the patient perspective. After the implementation of monetary incentives, the main positive feedback of patients was also that the doctor’s examination was very serious, and the doctor was very patient when answering questions. For a country lacking medical resources, this requires physicians to provide as much perceptible care as possible within a limited time in practical work.

One may argue that the short-term effect cannot be inferred due to the maximum of patient satisfaction being 100 points. However, the period of the effects in Figure 2 shows that the influence of performance pay could be identified after the first payment of performance pay, and the estimation of the effect of the second payment was quite similar to the first time. Moreover, the effect on the efforts that we focused on showed the same pattern. Thus, we can infer that the patient satisfaction dropped after the payment. However, considering the intercept, patient satisfaction should not be too low even if the performance pay has a short-term effect.

Another piece of evidence for the short-term effect of performance pay is that, despite the fact that employees participated in a performance-setting meeting in the early stage, the ESA results showed that this meeting did not elicit any policy effect, even though the outcome of the meeting established incentive expectations. The failure of incentive expectations also shows that, for incentive policies, especially in the public sector, expectation management is not as good as a bird in the hand.

Therefore, from the perspective of patient satisfaction, the role of monetary incentives is mainly short-term and, thus, the question is how to build a patient-centered hospital culture within the hospital. However, the short-term role also has its advantages. As mentioned above, the physicians would put effort into the three dimensions to improve patients’ satisfaction. During the COVID-19 pandemic, medical staff are under significant occupational pressure, and monetary incentives can not only promote patients’ satisfaction and alleviate the current stage of doctor–patient conflict caused by the shortage of medical resource, but also improve the salary level of medical staff through monetary incentives, which usually cannot be modified due to policy governance, and promote physicians’ work satisfaction [16,26].

The research method in this paper expanded the scope of use of DiD. DiD is an effective policy identification method, and setting the control group and the treatment group is the core of identification [27]. However, the setting of the control group and the treatment group is impossible when the policy is implemented across a whole entity. This study extended the method of grouping by individual characteristics [28,29] and introduced it from financial research to hospital management. The segmentation group results in this paper were based on the revenue composition of hospital departments and the elasticity of the services that departments provided. Departments in which patient satisfaction can directly determine their income were classified as control groups, as the incentives had been implemented in the past through patient treatment-seeking behavior, and the new incentives did not affect the characteristics of the departments.

Based on the above discussion, we believe that future research can focus on the following aspects. The first is to conduct experiments on incentive policies in more hospitals to further strengthen the evidence. The second is to study how to motivate medical staff to be patient-centered in the long term. Medical services should be provided and patient satisfaction improved in the long-term. Finally, research should investigate how to build a patient-centered hospital culture in the hospital and strengthen the effects of incentive policies.

## 5. Conclusions

The aim of the present research was to examine the effect of monetary incentives on physicians’ efforts towards ensuring patients’ satisfaction. To implement the research, we divided the study into two parts. Firstly, we used DiD estimation to test whether monetary incentives could promote patient satisfaction. We found that the patient satisfaction was significantly improved by 2.7905 points (*p* < 0.001). The parallel trend assumption was tested using the ESA method. Secondly, we tested the conceptual framework using two-stage regression and the ESA method. The empirical results showed that, even if specific measures were not introduced to physicians, they would still proactively enhance the patient experience by protecting patient privacy, allowing patients to feel motivated by their work, and improving doctor–patient communication.

## 6. Limitation

The main limitation of this paper was that the sample size was not large enough, and the ideal study should carry out an investigation and intervention in multiple hospitals, including secondary and tertiary hospitals; however, considering the research period of this study and the number of patients investigated, this limitation is difficult to overcome. Using the calibration method from HCAHPS^®^, we tried to calibrate the bias of the score, but the bias may still have existed.

Patient satisfaction is an important topic in public health and in the management of hospital, so we also recommend that more human resources intervention research be carried out in the future.

## Figures and Tables

**Table 1:** Ranking of performance bonus and salary ratio.

Department	Ratio	Ranking	Groups
Dermatology	1.957	1	Control
Dentistry	1.921	2	Control
Pediatrics	1.597	3	-
Ear, nose and throat	1.450	4	-
Pain medicine	1.420	5	-
Internal medicine	1.292	6	Treat
Obstetrics/gynecology	1.239	7	Treat

**Table 2:** Summary statistics of the variables.

	N	Mean	SD	Min	25%	Median	75%	Max
Age	994	44.36	19.28	3	29	38	60	95
Gender (female = 0)	994	0.34	0.48	0	0	0	1	1
Non-resident (yes = 1)	994	0.46	0.49	0	0	0	1	1
Education	994	0.98	1.12	0	0	1	2	4
Hospital satisfaction	994	95.14	7.27	50	90	100	100	100
Physician satisfaction	994	95.61	6.94	50	90	100	100	100
Communication	994	96.748	6.52	50	95	100	100	100
Privacy protection	994	97.38	5.56	60	98	100	100	100
Working effort	994	96.51	6.86	50	95	100	100	100
Treat	994	0.84	0.36	0	1	1	1	1
Post	994	0.38	0.49	0	0	0	1	1
DiD	994	0.34	0.47	0	0	0	1	1

**Table 3:** Difference-in-difference of physician satisfaction.

	Physician Satisfaction	Communication	Working	Privacy Protection	Physician Satisfaction
DiD	2.7905	1.6614	1.0416	1.0997	1.6309
	(0.0000)	(0.0000)	(0.0349)	(0.0004)	(0.0000)
Age	0.0117	0.0093	−0.0088	0.0064	0.0107
	(0.1106)	(0.1908)	(0.2453)	(0.3717)	(0.0334)
Education	−0.5040	−0.5086	−0.4851	−0.2676	−0.1090
	(0.0000)	(0.0012)	(0.0000)	(0.0080)	(0.0543)
Non-resident	−0.2307	−0.1234	−0.7805	−0.4937	0.2581
	(0.4387)	(0.7798)	(0.0017)	(0.1712)	(0.1633)
Gender	0.7935	0.8245	0.7405	1.1465	−0.0661
	(0.0070)	(0.0876)	(0.1830)	(0.0040)	(0.7787)
Communication					0.2410
					(0.0015)
Working					0.3782
					(0.0000)
Privacy protection					0.3322
					(0.0000)
Intercept	94.484	96.051	97.136	96.823	2.4368
	(0.0000)	(0.0000)	(0.0000)	(0.0000)	(0.4446)
Fixed effect	Control	Control	Control	Control	Control
Time effect	Control	Control	Control	Control	Control
R-squared	0.130	0.119	0.085	0.126	0.595
No. of observations	994	994	994	994	994

Note: The values in parentheses are the *p*-values under cluster standard errors clustered by department.

**Table 4:** Difference-in-difference of hospital satisfaction.

	Hospital Satisfaction	Communication	Working	Privacy Protection	Hospital Satisfaction
DiD	2.7920	1.6614	1.0416	1.0997	1.5594
	(0.0000)	(0.0000)	(0.0349)	(0.0004)	(0.0000)
Age	0.0144	0.0093	−0.0088	0.0064	0.0129
	(0.1098)	(0.1908)	(0.2453)	(0.3717)	(0.1051)
Education	−0.4955	−0.5086	−0.4851	−0.2676	−0.0759
	(0.0004)	(0.0012)	(0.0000)	(0.0080)	(0.3054)
Non-resident	−0.2311	−0.1234	−0.7805	−0.4937	0.2341
	(0.4274)	(0.7798)	(0.0017)	(0.1712)	(0.1652)
Gender	1.0108	0.8245	0.7405	1.1465	0.1500
	(0.0282)	(0.0876)	(0.1830)	(0.0040)	(0.5220)
Communication					0.3271
					(0.0000)
Working					0.3689
					(0.0000)
Privacy protection					0.2774
					(0.0000)
Intercept	93.805	96.051	97.136	96.823	−0.3016
	(0.0000)	(0.0000)	(0.0000)	(0.0000)	(0.9182)
Fixed effect	Control	Control	Control	Control	Control
Time effect	Control	Control	Control	Control	Control
R-squared	0.135	0.119	0.085	0.126	0.582
No. of observations	994	994	994	994	994

Note: The values in parentheses are the *p*-values under cluster standard errors clustered by department.

## Data Availability

Not applicable.
